# First-principles theoretical study of hydrolysis of stepped and kinked Ga-terminated GaN surfaces

**DOI:** 10.1186/1556-276X-8-232

**Published:** 2013-05-16

**Authors:** Mari Oue, Kouji Inagaki, Kazuto Yamauchi, Yoshitada Morikawa

**Affiliations:** 1 Department of Precision Science and Technology, Graduate School of Engineering, Osaka University, 2-1 Yamada-oka, Suita, Osaka 565-0871, Japan; 2 Research Center for Ultra-Precision Science and Technology, Graduate School of Engineering, Osaka University, 2-1 Yamada-oka, Suita, Osaka 565-0871, Japan

**Keywords:** GaN surfaces, Hydrolysis, CARE method, Step-terrace structure, DFT

## Abstract

We have investigated the initial stage of hydrolysis process of Ga-terminated GaN surfaces by using first-principles theoretical calculations. We found that the activation barrier of H_2_O dissociation at the kinked site of the Ga-terminated GaN surface is about 0.8 eV, which is significantly lower than that at the stepped site of about 1.2 eV. This is consistent with the experimental observation where a step-terrace structure is observed after the etching process of Ga-terminated GaN surfaces with catalyst-referred etching method. Detailed analysis on the nature of the chemical interaction uring the hydrolysis processes will be discussed.

## Background

GaN has been attracting enormous attention because it is one of the most promising materials for short-wavelength optoelectronic devices such as light-emitting diodes, blue laser diodes, and high-power, high-frequency electronic devices [1,2]. The performance of these semiconductor devices depends on the quality of GaN crystals, and it is important to prepare atomically smooth, damage-free surfaces for homoepitaxial growth of high-quality GaN layers. Recently, catalyst-referred etching (CARE) has been proposed as a new finishing method. By using this method, atomically smooth surfaces with step-terrace structure were obtained [3-5]. GaN surfaces can be etched even by pure water with Pt as a catalyst [6,7]. However, the remaining problem in this method is its low removal rate. To find a clue on how to improve the removal rate, it is important to clarify the etching process at the atomic level and find determinant factors in the process. Because step-terrace surfaces were observed in the CARE-processed surfaces, the etching reactions at step edges are considered to be important. In this paper, we analyzed elementary reaction processes and their activation barriers of dissociative adsorption of water and hydrolysis of Ga-terminated GaN surfaces as the initial stage of etching processes by means of first-principles calculations.

## Methods

### Calculation method and model

All calculations were performed using STATE program package [8] based on density functional theory within a generalized gradient approximation, and we employed an exchange-correlation energy functional proposed by Perdew et al. [9]. We used ultrasoft pseudopotentials to describe the electron-ion interactions [10]. Wave functions are expanded by a plane-wave basis set, and cut-off energies for wave function and charge density are set to be 25 and 225 Ry, respectively. The reaction barriers of dissociative adsorption of water are calculated by a climbing image nudged elastic band (NEB) method [11].

**Figure 1:**
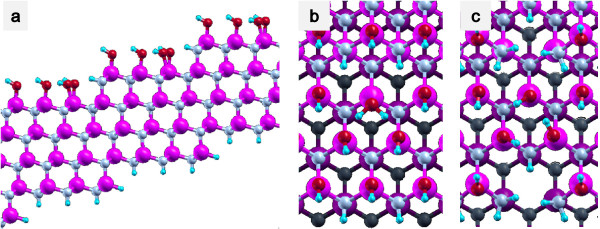
**Calculation model.** (**a**) Side view and (**b**) top view of a step-terrace structure. (**c**) Top view of a kinked structure.

Since experimentally observed surface consists of step-and-terrace surface atomic structure, we investigated hydrolysis processes at stepped GaN surfaces using a repeated slab model. GaN has wurtzite structure as its most stable crystal structure. If the Ga-terminated GaN(0001) surface is inclined towards the $\langle 1\bar{1}00\rangle$ direction, two types of steps appear alternatively, and to model an inclined GaN(0001) surface by using the repeated slab model, we have to include two steps in a unit cell. Instead, we employed a zinc blende GaN(221) surface as shown in Figure 1, where only one type of step is included and the size of the unit cell can be reduced by half compared with the wurtzite substrate. Due to the small energy difference between wurtzite and zinc blende structure (0.014 eV), we assume that the reactivity of the two surfaces are very close to each other. Our slab model consists of four GaN bilayers as shown in Figure 1. We also investigated hydrolysis processes at kinked sites. Figure 1b indicates an ordinary step-terrace structure, and Figure 1c indicates a kink-like structure. However, the ‘kink-like structure’ here does not represent a proper kinked structure. In this structure, one out of every two Ga atoms is removed from a step, and N dangling bonds are terminated by H atoms. Thus, the present kink-like structure has higher reactivity than ordinary kinked structures, and the reactivity of true kink sites may be in between those of the present kink-like structure and the step structure. The work function difference between the two surfaces of a slab is compensated by an effective screening medium method proposed by Otani and Sugino [12]. Dangling bonds at the bottom layers of N and Ga atoms are terminated by pseudo-hydrogen atoms which have fractional number of nuclear charges, i.e., a hydrogen with atomic number of 0.75 to terminate a dangling bond of N and a hydrogen with atomic number of 1.25 to terminate a dangling bond of Ga.

## Results and discussions

### Termination of the GaN surface

**Figure 2:**
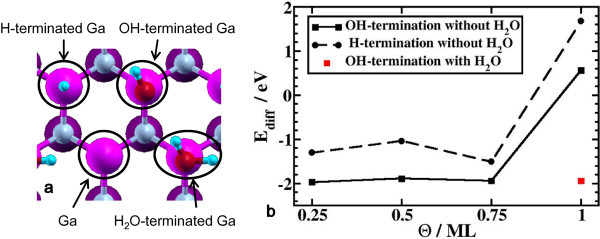
**Geometries and differential adsorption energies of H, OH, and H**_**2**_**O on a GaN surface.** (**a**) Top view of H, OH, and H_2_O on a zinc blende GaN(111) surface. (**b**) Differential adsorption energy of OH (black square) and H (black circle) as a function of surface coverage *Θ*. The differential adsorption energy of H_2_O on 0.75 ML of OH-terminated surfaces is also shown by a red square.

Before investigating dissociative adsorption processes of H_2_O molecule, we examined the termination of surface Ga atoms. Since the etching reaction occurs in pure water with Pt plate in contact with GaN surface, surface Ga atoms are considered to be terminated by H atoms or OH groups (see Figure 2a). We calculated the differential heat of adsorption of H and OH as a function of surface coverage. The results are shown in Figure 2b. The formation energies of H-terminated (*E*_*f*_[H_*n*_/GaN]) and OH-terminated (*E*_*f*_[(OH)_n/GaN]) surfaces are calculated by Equations 1 and 2:

(1) $$\begin{array}{lll} E_{f}[\;\text{H/GaN}](\Theta ) & =E[\;{\text{H}}_{n}\text{/GaN}]\; & \;\\ & \;\;\;\;-\left(E[\;\text{GaN}]+\frac{n}{2}E[\;{\text{H}}_{2}]\right)\; \end{array}$$

(2) $$\begin{array}{lll} \;E_{f}[\;\text{(OH)/GaN}](\Theta ) & =E[\;{\text{(OH)}}_{n}\text{/GaN}]\; & \;\\ & \;\;\;\;-\left(E[\;\text{GaN}]+\text{nE}[\;{\text{H}}_{2}\text{O}]-\frac{n}{2}E[\;{\text{H}}_{2}]\right)\; \end{array}$$

where *E*[ GaN] is the total energy of a GaN(111) 2×2 surface unit cell, *Θ* is the coverage of H (or OH) defined by *n*/4, and *n* is the number of adsorbed H or OH in the GaN(111) 2×2 surface unit cell. By taking the derivative of the formation energies with respect to the surface coverage, we calculated the differential adsorption energies of H and OH as a function of surface coverage.

(3) $$\begin{array}{l} \;E_{\text{diff}}[\;\text{H/GaN}](\Theta )\equiv \frac{d}{\mathrm{d\Theta}}E_{f}[\;\text{H/GaN}](\Theta )\;\\ \;=E_{f}[\;{\text{H}}_{n}\text{/GaN}]-E_{f}[\;{\text{H}}_{n-1}\text{/GaN}]\; \end{array}$$

(4) $$\begin{array}{l} \;E_{\text{diff}}[\;\text{(OH)/GaN}](\Theta )\equiv \frac{d}{\mathrm{d\Theta}}E_{f}[\;\text{(OH)/GaN}](\Theta )\;\\ \;=E_{f}[\;{\text{(OH)}}_{n}\text{/GaN}]-E_{f}[\;{\text{(OH)}}_{n-1}\text{/GaN}]\; \end{array}$$

Figure 2b shows that OH termination is more stable than H termination for all coverages. Moreover, the differential adsorption energy becomes positive for *Θ*>0.75 ML for both H and OH termination. This can be understood by counting the number of electrons in the surface dangling bonds. Each surface Ga atom has one dangling bond, and on average, three-fourth of the electrons are accommodated in each dangling bond. Therefore, if the coverage of H or OH is 0.75 ML, their dangling bonds are fully occupied by paired electrons, and the remaining 25% of surface dangling bonds become empty, forming a closed-shell electronic structure. A closed-shell electronic structure can be also formed by terminating the remaining 25% dangling bonds with H_2_O. As seen in Figure 2b, the differential adsorption energy of H_2_O is −1.93 eV, further stabilizing the OH-terminated GaN surface. An empty Ga dangling bond attracts the lone pairs of H_2_O as observed at the water/GaN(10$\bar{1}$0) interface [13]. Therefore, in the following calculations, we terminated 75% of surface Ga dangling bonds with OH and 25% with H_2_O.

### Dissociative adsorption of H_2_O

**Figure 3:**
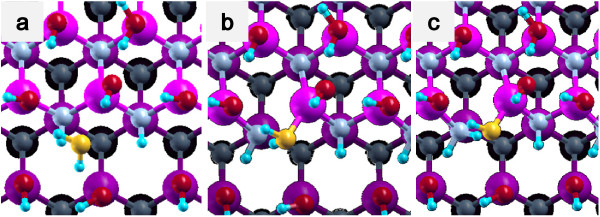
**Side bond process in a step-terrace structure.** (**a**) Initial state, (**b**) transition state, and (**c**) final state.

**Figure 4:**
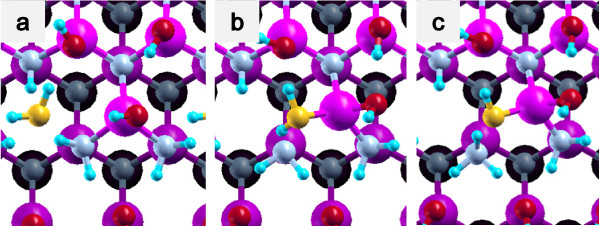
**Side bond process in a kinked structure.** (**a**) Initial state, (**b**) transition state, and (**c**) final state.

**Figure 5:**
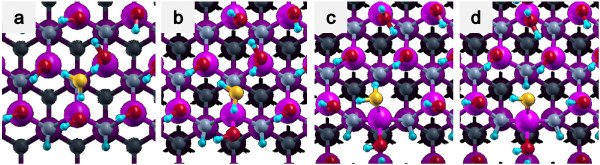
**Back bond process in a step-terrace structure.** (**a**) Initial state, (**b**) first transition state (**c**) second transition state, (**d**) final state.

**Figure 6:**
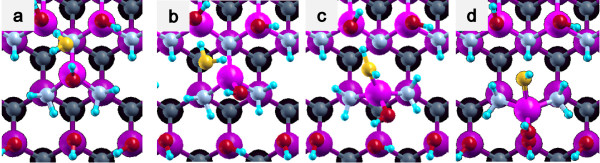
**Back bond process in a kinked structure.** (**a**) Initial state, (**b**) first transition state, (**c**) second transition state, and (**d**) final state.

We investigated two possible dissociative adsorption paths of H_2_O at stepped and kinked sites of Ga-terminated GaN surfaces as follows: 

(1) Side bond process: OH of a H_2_O molecule is bound to Ga at a step edge, and the remaining H of a water molecule is bound to N at a step edge (Figures 3c and 4c).

(2) Back bond process: OH is bound to Ga at a step edge, and the remaining H is bound to N at terrace (Figures 5d and 6d).

**Figure 7:**
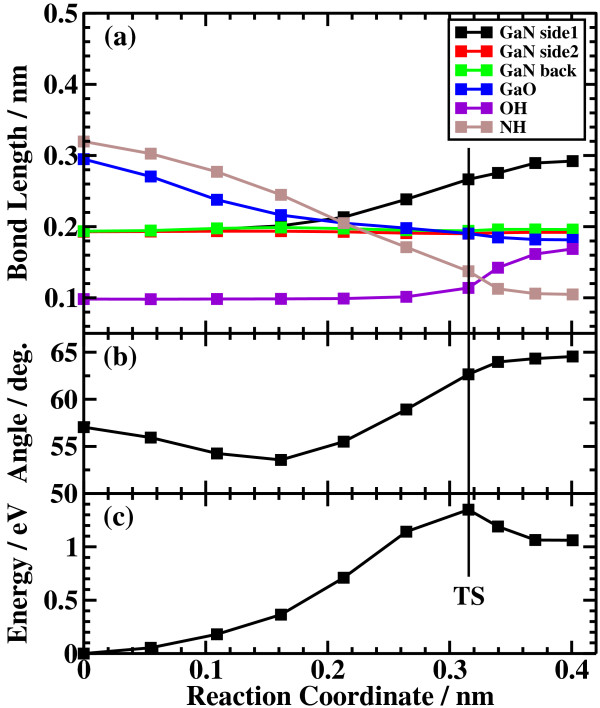
**Results of the side bond process at the step structure.** (**a**) Bond length, (**b**) dihedral angle of Ga-N-Ga-N, and (**c**) energy profiles of the side bond process at the step structure.

**Figure 8:**
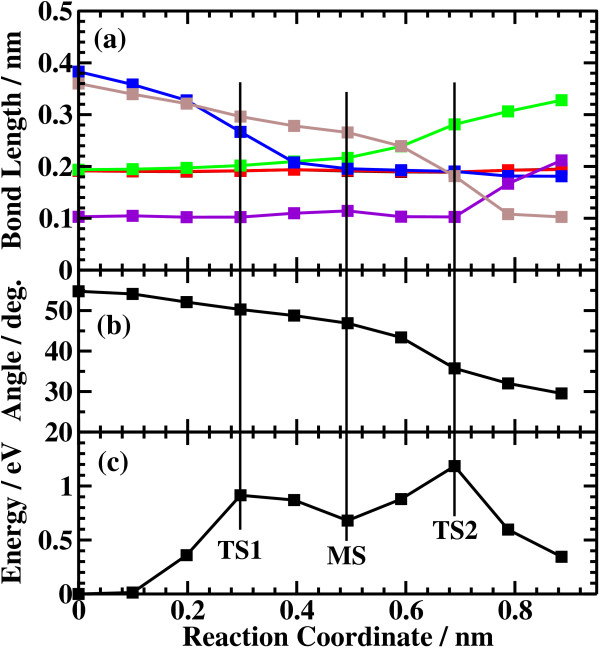
**Results of the back bond process at the step structure.** (**a**) Bond length, (**b**) dihedral angle of Ga-N-Ga-N, and (**c**) energy profiles of the back bond process at the step structure.

**Figure 9:**
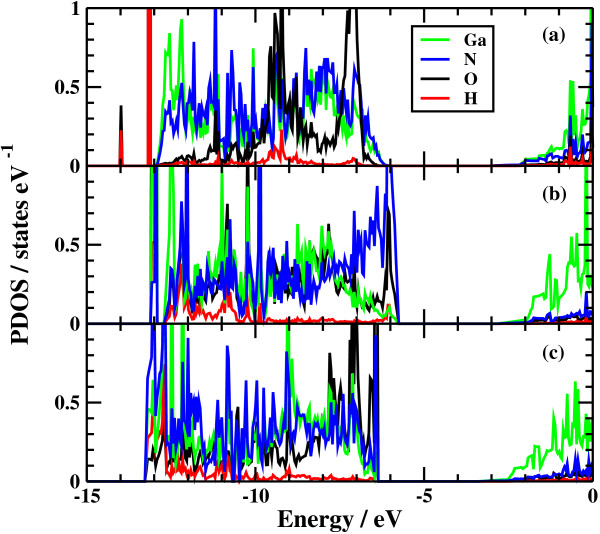
**Projected density of states of the side bond process at the step-terrace structure.** (**a**) Initial state, (**b**) transition state, and (**c**) final state. Peak shift of N 2*p* and O 2*p* indicates the dissociation of Ga-N bond.

**Figure 10:**
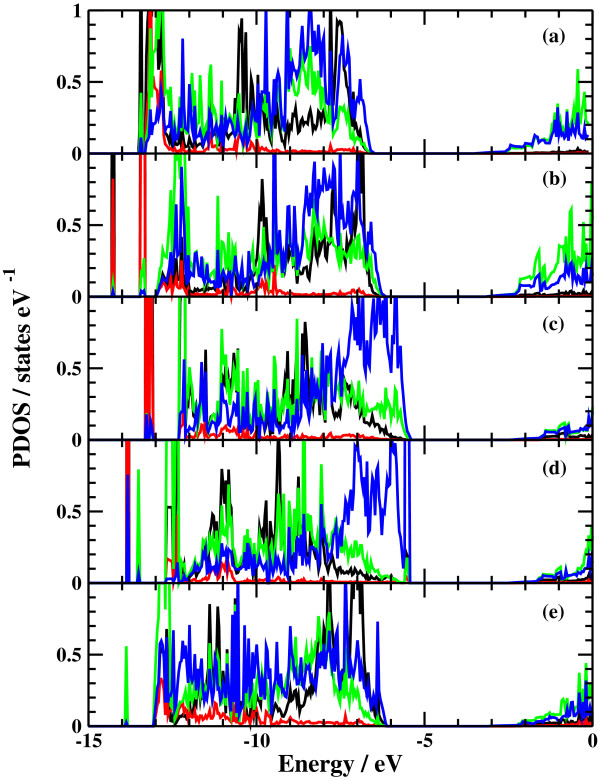
**Projected density of states of the back bond process at the step-terrace structure.** (**a**) Initial state, (**b**) first transition state, (**c**) intermediate state, (**d**) second transition state, and (**e**) final state.

**Figure 11:**
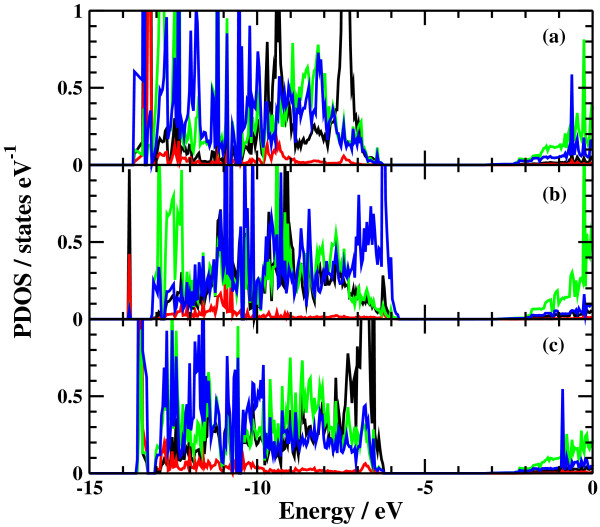
**Projected density of states of the side bond process at the kinked structure.** (**a**) Initial state (**b**) transition state, and (**c**) final state.

**Figure 12:**
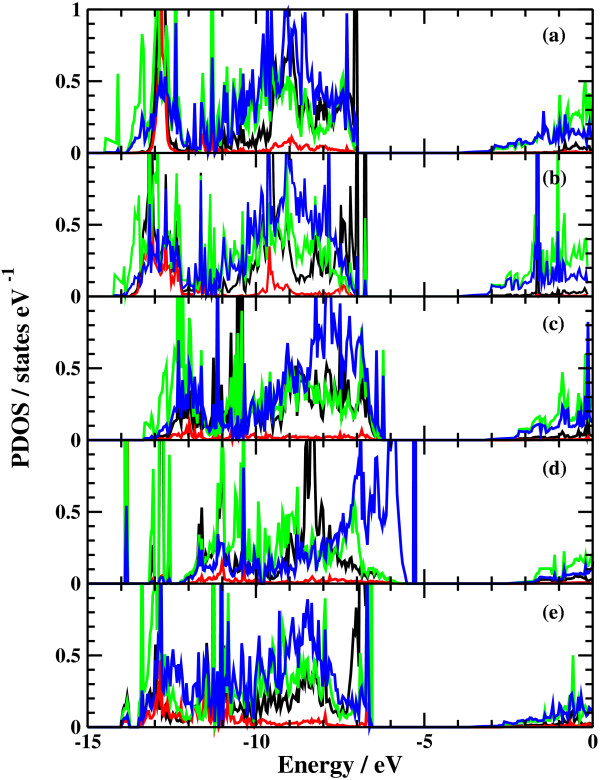
**Projected density of states of the back bond process at the kinked structure.** (**a**) Initial state, (**b**) first transition state, (**c**) intermediate state, (**d**) second transition state, and (**e**) final state.

The potential energy profiles for the side bond process and the back bond process in a step-terrace structure are shown in Figures 7c and 8c as a function of reaction coordinate *S*. Here, the reaction coordinate *S* is defined by the distance along the minimum energy path obtained by the NEB method in the multidimensional configuration space. The side bond process has one transition state, and its reaction barrier is 1.35 eV. Figure 3 shows the atomic structures of the initial state, transition state, and final state of the side bond process. The back bond process has two transition states (Figure 5b,c), and its reaction barrier is 1.18 eV as seen in Figure 8c. Surface structures of the initial state, the first transition state, the second transition state, and the final state of the side bond process are shown in Figure 5. The bond lengths for the side bond and the back bond processes at the step-terrace structure are shown in Figures 7a and 8a, respectively. The positions of transition states are indicated by vertical lines. In the early stage of the side bond process (*S*≤0.2 nm), a water molecule approaches a surface Ga-N bond, and bond lengths of *r*(Ga-O) and *r*(N-H) are reduced, while no bonds are broken. Therefore, the energy increase from *S*=0 to 0.2 nm is mainly due to the Pauli repulsion between H_2_O and the surface GaN bond. At *S*≃0.2 nm, the Ga-N bond starts breaking, and the energy is further increased. After the transition state, i.e., *S*≃0.32 nm, the bond switching from O-H bond to N-H bond takes place. Similarly, in the case of the back bond process, before the first transition state (0 nm ≤*S*≤0.3 nm), a water molecule approaches the surface Ga-N bond. Between the two transition states (0.32 nm ≤*S*≤0.68 nm), the bond switching from GaN to GaO takes place, and after the second transition, the bond switching from O-H to N-H takes place. To further confirm the electronic origin of the potential energy profile, we have calculated the projected density of states (PDOS) onto atomic orbitals, and the results are shown in Figures 9, 10, 11, and 12. Figure 9 shows the PDOS for the initial, the transition, and the final states of the side bond process at the step-terrace structure. In the figure, the abscissa indicates the energy with the energy zero taken at the vacuum level, and the ordinate indicates the density of states. In the initial state, the N 2*p* state is broadly distributed from −6.2 to −13 eV, and the O 2*p* state has a sharp peak close to the valence top, i.e., at around −7.0 eV. In the transition state, N 2*p* state has a sharp peak at the top of the valence band located at around −5.8 eV, indicating the dissociation of Ga-N bond. Figure 10 shows the PDOS onto atomic orbitals for the initial, the first transition, the intermediate, the second transition, and the final states of the back bond process at the step-terrace structure. In the initial state, the N 2*p* state is broadly distributed from −6.6 to −13.5 eV, and the O 2*p* state has a peak at around −7.5 eV. On going from the initial to the second transition states, the N 2*p* state shifted continuously towards lower binding energy to the top of the valence band, while the O 2*p* state shifted to lower binding energy up to the first transition state and then shifted to higher binding energy after the first transition state. At the second transition state, the N 2*p* state has a sharp peak at the top of the valence band, i.e., located at around −5.5 eV (Figure 10d), indicating the breaking of Ga-N bond. Therefore, the energy increase at the first transition state can be ascribed to the Pauli repulsion between the saturated H_2_O and G-N bonds, and that at the second transition state can be ascribed to the bond switching from Ga-N and O-H bonds to Ga-O and N-H bonds.

**Figure 13:**
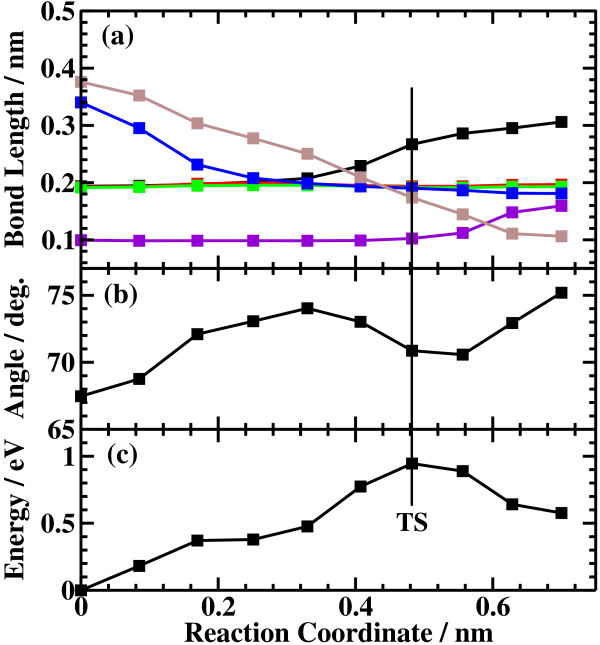
**Results of the side bond process at the kinked structure.** (**a**) Bond length, (**b**) dihedral angle of Ga-N-Ga-N, and (**c**) energy profiles of the side bond process at the kinked structure.

**Figure 14:**
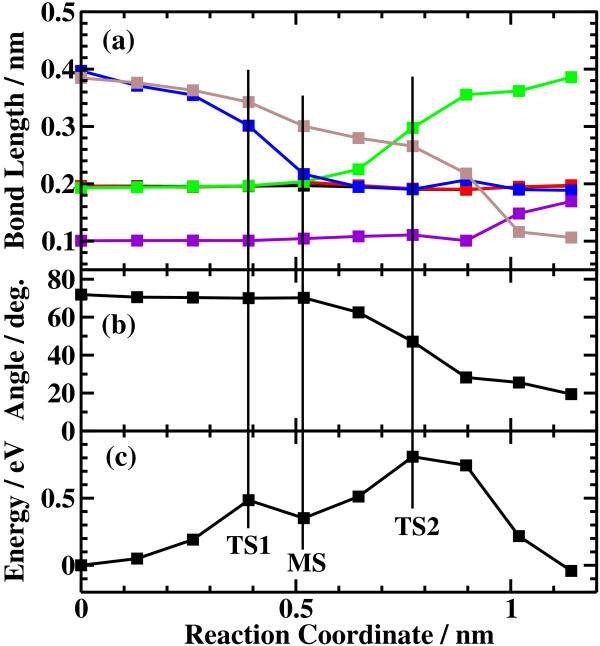
**Results of the back bond process at the kinked structure.** (**a**) Bond length, (**b**) dihedral angle of Ga-N-Ga-N, and (**c**) energy profiles of the back bond process at the kinked structure.

The potential energy profiles of the side bond process and the back bond process in the kinked structure are shown in Figures 13c and 14c, respectively. Similar to the step-terrace structure, the side bond process has one transition state (Figure 4b), and the back process has two transition states (Figure 6b,c). The reaction barriers for the side bond and the back bond processes are 0.95 and 0.81 eV, respectively (see Figures 13c and 14c). The bond lengths for the side bond and the back bond processes at the kinked structure as a function of reaction coordinate *S* are shown in Figures 13a and 14a, respectively. The results are similar to those for the step-terrace structure, and the energy increase in the early state of the reaction path is attributed to the Pauli repulsion between a closed-shell water molecule and a surface Ga-N bond, while one in the latter half of the reaction path is attributed to the bond switching from Ga-N and O-H bonds to Ga-O and N-H bonds.

The barrier heights and the energies of the final states relative to the initial states for the four processes are summarized in Table 1. In the case of back bond process, the barrier heights are systematically lower and the final states are more stable compared with the case of the side bond processes. The reason why the dissociative adsorption of H_2_O occurs more easily in the back bond process than in the side bond process can be understood as follows: In the case of the side bond process, when a Ga-N bond is broken and H_2_O is dissociatively adsorbed, the Ga atom moves towards the upper terrace. However, the nearest neighboring N atoms are bound to the next nearest Ga atoms, and their movement is restricted, strongly hindering the relaxation of the Ga atom towards the upper terrace site. On the other hand, in the back bond process, Ga atom bound to OH can relax towards the lower terrace significantly because the nearest neighboring N is only bound to one next nearest Ga atom, allowing the relaxation of Ga towards the lower terrace site. The magnitude of the geometric relaxation of surface Ga can be seen from the dihedral angle of Ga(step edge)-N(step edge)-Ga(second layer)-N(second layer) shown in Figures 7b, 8b, 13b, and 14b. As seen in Figures 7b and 13b, the dihedral angle is changed by about only 10° during the reaction for the case of the side bond processes. On the other hand, for the case of the back bond process, the dihedral angle is changed by as large as 35° for the case of step-terrace site and 50° for the case of kink site.

**Table 1:** Barrier height and the energy of the final state relative to the initial state

		**Barrier** **height/eV**	**Energy** **difference/eV**	
Step-terrace structure	Side bond	1.35	1.06	
	Back bond	1.18	0.34	
Kinked structure	Side bond	0.95	0.58	
	Back bond	0.81	−0.04	

It is found that the dissociative adsorption of water in the back bond process at the kinked structure is the most energetically favorable path we have investigated so far. Therefore, we think that etching reactions take place predominantly at kinked sites. Note that our kinked model represents an extreme case, and the activation barriers of dissociative adsorption of H_2_O should be somewhat larger than our calculated values but still smaller than those calculated for stepped sites.

Before closing our discussion, we mention about roles of additional water molecules terminating empty Ga dangling bonds. As discussed above, 75% of surface Ga dangling bonds are terminated by OH and 25% are by H_2_O. These additional H_2_O molecules initiate proton transfer on the GaN surfaces and promote chemical reactions at surfaces as discussed by Wang and co-workers [13]. Actually, additional water molecules play an active role in two step processes of H_2_O dissociation, in which H_2_O molecule is dissociated, OH is bound to surface Ga, and H is bound to neighboring H_2_O (MO et al., unpublished results). Following this process, proton transfer takes place to terminate a dangling bond at subsurface N. However, in the direct H_2_O dissociation we have investigated in the present study, it seems that the additional water molecules are spectator of the reaction, and they play a rather minor role.

## Conclusions

In summary, we have investigated the initial stage of hydrolysis process of Ga-terminated GaN surfaces by using first-principles theoretical calculations. The activation barrier of H_2_O dissociation at kinked sites of the Ga-terminated GaN(0001) surface is about 0.8 eV, which is significantly lower than that at stepped sites of about 1.2 eV, suggesting that etching reactions take place predominantly at kinked sites of GaN surfaces; and this is consistent with the experimental observation where a step-terrace structure is observed after the etching process of Ga-terminated GaN(0001) surfaces with CARE method. The origin for the activation barriers are ascribed to the Pauli repulsion in the early stages of hydrolysis process, while they are ascribed to the bond switching between OH bond of H_2_O and NH bond at the edge of a stepped site.

## Competing interests

The authors declare that they have no competing interests.

## Authors’ contributions

MO carried out the theoretical work in collaboration with KI. KY supplied experimental information. YM is the supervisor of the project. All authors read and approved the final manuscript.
